# Extracellular defense of bacteria against antimicrobial peptides

**DOI:** 10.1128/jb.00166-25

**Published:** 2025-08-01

**Authors:** Osmel Fleitas, Eria A. Rebollar, Víctor H. Bustamante

**Affiliations:** 1 Departamento de Microbiología Molecular, Instituto de Biotecnología, Universidad Nacional Autónoma de México 42560, Cuernavaca, Morelos, México; 2 Programa de Microbiología Genómica, Centro de Ciencias Genómicas, Universidad Nacional Autónoma de México 61740 , Cuernavaca, Morelos, México; University of Southern California, Los Angeles, California, USA

**Keywords:** extracellular defense, bacterial resistance, bacterial defense, AMPs, antimicrobial peptides

## Abstract

Antimicrobial peptides (AMPs) are short chains of amino acids naturally produced by all kingdoms of life, which exhibit broad-spectrum activity against bacteria, fungi, and viruses and thus play a crucial defense role in organisms. Unlike conventional antibiotics, AMPs are less prone to induce bacterial resistance since they can act on multiple targets, mainly affecting cell membranes. Thus, AMPs are considered promising antibiotic agents for medical applications. However, bacteria have developed different mechanisms to resist the action of AMPs, which operate at the extracellular, surface, and intracellular levels. Extracellular defense against AMPs is mediated by an arsenal of molecules or cell-derived particles or structures that are secreted and constitute the bacterial releasome. The bacterial releasome-associated factors can sequester, degrade, or chemically modify AMPs, thus providing individual and collective bacterial defense against AMPs. This minireview describes how diverse and impressive the releasome mechanisms mediating AMPs resistance are as a first line of defense.

## INTRODUCTION

Antimicrobial peptides (AMPs) are short amphipathic peptides that are naturally produced by all kingdoms of life and constitute a defense system in organisms against pathogenic microorganisms (1–3). The AMPs are typically between 12 and 50 amino acids in length, contain a high proportion of hydrophobic residues (~50%), and are typically cationic at physiological pH (net positive charge of 2^+^−11^+^) (4, 5). They exhibit a wide variety of structures, which can be formed by α-helices, β-sheets, linear regions, or more complex structural arrangements (e.g., cyclic structures) (6).

**Fig 1:**
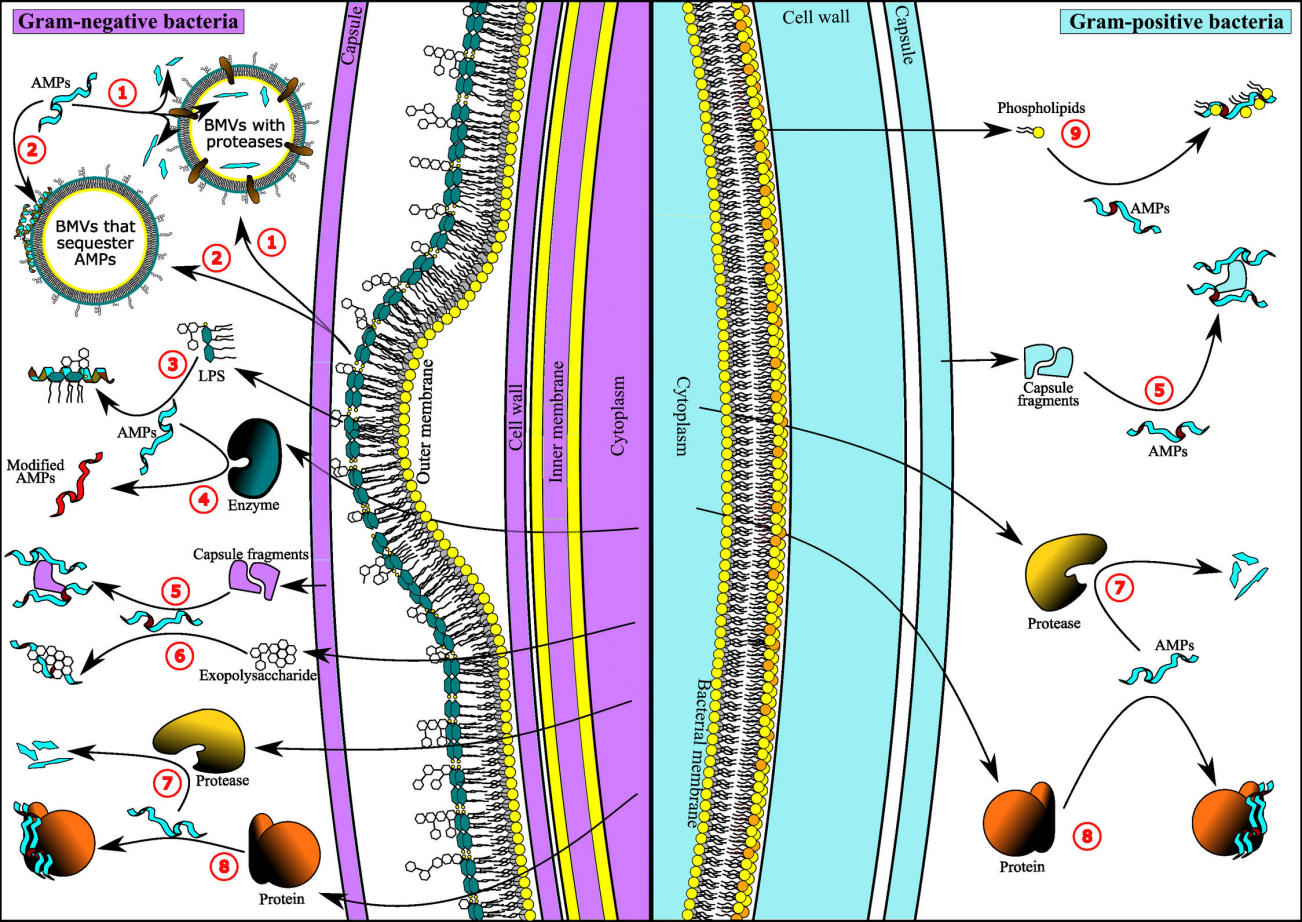
Extracellular defense mechanisms of bacteria against AMPs. Mechanisms present in both Gram-negative and Gram-positive bacteria are shown. (1) Released bacterial membrane vesicles (BMVs) containing proteases degraded AMPs. (2) Released BMVs sequester AMPs through interactions with LPS and proteins contained in the BMVs. (3) Released LPSs bind and sequester AMPs. (4) Secreted enzymes chemically modify AMPs. (5) Released capsular fragments bind and sequester AMPs. (6) Released exopolysaccharides bind and sequester AMPs. (7) Secreted proteases degrade AMPs. (8) Secreted proteins bind and sequester AMPs. (9) Released phospholipids bind and sequester AMPs. This figure was generated by Dr. Harrys Morales Duque.

The AMPs exert their activity primarily by disrupting cell membranes through several mechanisms: the carpet, barrel-stave, toroidal pore, flood-gate mechanism, aggregate channel, lipid segregation into domains, among others (7, 8). However, AMPs can also act by inhibiting DNA replication, protein synthesis and folding, metabolic processes, protease activity, cell wall synthesis, and cell division (9, 10). The AMPs have attractive properties for use in therapy: a rapid and broad-spectrum microbicidal activity in the µM concentration range, are active against polymicrobial biofilms and metabolically dormant bacteria, can act synergistically with conventional antibiotics, and are considered less prone to develop resistance in bacteria (11–13). However, bacteria have developed a wide variety of mechanisms to overcome the activity of AMPs (14). These resistance mechanisms operate at the intracellular, surface, or extracellular levels. At the intracellular level, AMPs can be degraded by proteases or expelled to the cell exterior by efflux pumps (15, 16). At the surface level, AMPs can be prevented from binding and/or entering the cell through a series of modifications in the cell membranes and/or cell wall or by the presence of external physical barriers, such as the capsule and the S-layer (16–18); in addition, AMPs can be degraded by proteases or sequestered by biomolecules anchored to the cell surface (19, 20). At the extracellular level, AMPs can be degraded, sequestered, or chemically modified by various molecules secreted by bacteria (21–23). The collection of molecules (proteins, exopolysaccharides, nucleic acids, and metabolites) and membrane vesicles released by bacteria into the extracellular environment has been denominated as the bacterial “releasome” (24). Recent experimental evidence supports the critical importance of the releasome as the first line of defense in bacteria, mediating individual and collective protection against AMPs (25–27). Some studies support that the protection offered by releasome-associated mechanisms against AMPs is transient (28, 29), which could be due to the high energetic cost of maintaining active production and secretion of molecules. Thus, releasome-associated protection seems to operate as a short-term first line of defense. This minireview describes the different mechanisms mediated by the releasome to protect bacteria against the action of AMPs (Fig. 1; Table 1). To note, releasome-associated protective mechanisms also include the manipulation of host signaling pathways to suppress the production of AMPs (30–34), which is a field not included in this minireview.

**TABLE 1:** Bacterial releasome-associated defense mechanisms against AMPs

Defense mechanisms	Effectors	Bacteria	Target AMPs	References
Sequestration	Phospholipids	*S. aureus* *S. epidermidis* *S. pyogenes* *S. agalactiae* *S. gordonii* *E. faecalis*	Daptomycin Nisin Melittin	(28, 35)
	Exopolysaccharides	*P. aeruginosa* *B. cepacian* *I. limosus*	LL-37 SMAP-29 LL-37 (*Pongo pygmaeus*) LL-37 (*Presbitys obscurus*)	(36, 37)
	Capsular polysaccharides	*K. pneumoniae* *S. pneumoniae* *P. aeruginosa* *B. anthracis* *B. pertussis*	LL-37 SMAP-29 Polymyxin B hNP-1 hBD-2 hBD-3	(21, 37–39)
	Lipopolysaccharides	*P. aeruginosa* *E. coli* *A. baumannii*	Colistin	(40)
	Lipocalin BcnA	*B. cenocepacia* *P. aeruginosa* *M. tuberculosis* H37Rv *S. aureus* USA300	Polymyxin B	(41)
	Lactoferrin-binding protein B	*M. bovis*	Lactoferricin B LL-37	(42)
	Protein SIC	*S. pyogenes*	LL-37 hNP-1 hBD-1 hBD-2 hBD-3	(43, 44)
	Protein DRS	*S. pyogenes*	hBD-2 hBD-3 LL-37 hNP-1	(44, 45)
	Protein DrsG	*S. dysgalactiae* subsp. *equisimilis*	LL-37	(46)
	Protein staphylokinase	*S. aureus*	hNP-1 hNP-2	(47)
	Protein FAF	*F. magna*	hBD-3	(48)
	Extracellular DNA	*H. influenzae* *N. meningitidis* *S. epidermidis* *K. pneumoniae* *P. aeruginosa*	hBD-3 hBD-2 D-LL-31	(49–51)
	Bacterial membrane vesicles	*E. coli* *V. cholerae* *A. baumannii* *K. pneumoniae* *P. syringae* *B. bronchiseptica* *P. aeruginosa* *M. catarrhalis* *S. enterica* serovar Typhi	Polymyxin B Colistin LL-37 CATH-2 PMAP-36 Melittin	(29, 52–59)
AMP degradation	Endopeptidase O (PepO)	*S. suis*	LL-37 CRAMP	(23)
	Protease ApdS	*S. suis*	LL-37	(60)
	Colistin-degrading protease (Cdp)	*S. maltophilia*	Colistin	(27)
	Elastase	*P. aeruginosa*	LL-37	(61)
	Gelatinase	*E. faecalis*	LL-37	(61)
	ZapA	*P. mirabilis*	LL-37 hBD-1 Protegrin	(62)
	ZmpA	*B. cenocepacia*	LL-37 Protamine SLPI Elafin	(63)
	ZmpB	*B. cenocepacia*	Protamine SLPI Elafin hBD-1	(63)
	Aureolysin	*S. aureus*	LL-37	(64)
	Metalloproteases	*B. anthracis*	LL-37	(65)
	Gingipains	*P. gingivalis*	hBD-3	(66)
	SufA	*F. magna*	LL-37	(67)
	SepB	*S. pyogenes*	LL-37 hBD-2 hBD-3	(48, 61)
	PrtV	*V. cholerae*	Melittin LL-37	(68)
AMP modification	Porphyromonas peptidylarginine deiminase (PPAD)	*P. gingivalis*	LP-9	(22)

## SEQUESTRATION BY PHOSPHOLIPIDS

Sequestration of AMPs by bacterial decoys is one of the most studied defense mechanisms operating at the extracellular level (Fig. 1). For example, *Staphylococcus aureus* releases membrane phospholipids (mainly phosphatidylglycerol) that bind to and inactivate the peptides daptomycin, nisin, and melittin (28). Moreover, it was shown that physiological levels of phosphatidylglycerol protect *S. aureus* against daptomycin (28). The release of phospholipid decoys appears to be conserved (at least against daptomycin) in other Gram-positive pathogenic bacteria, such as *Enterococcus faecalis*, *Staphylococcus epidermidis*, *Streptococcus pyogenes*, *Streptococcus agalactiae,* and *Streptococcus gordonii* (35). Up to date, there is no experimental evidence showing this defense mechanism exists in Gram-negative bacteria.

## SEQUESTRATION BY EXOPOLYSACCHARIDES, LIPOPOLYSACCHARIDES, AND CAPSULAR FRAGMENTS

Bacteria can release exopolysaccharides (EPs), lipopolysaccharides (LPS), and capsular polysaccharides (CPs) that sequester and prevent the action of AMPs (Fig. 1; Table 1) (21, 36, 37, 40). The released EPs trap the AMPs within molecular complexes through electrostatic and hydrophobic interactions (36, 69, 70). EPs released by bacteria, such as *Pseudomonas aeruginosa*, *Inquilinus limosus*, and *Burkholderia cepacia*, interfered with the activity of several orthologous cathelicidin peptides, such as LL-37 produced by human, *ppy*LL-37 produced by *Pongo pygmaeus*, and *pob*RLL-37 produced by *Presbitys obscurus* (36). Similarly, the EPs alginate and cepacian released by *P. aeruginosa* and *B. cepacia*, respectively, and the CPs released by *Klebsiella pneumoniae* interfere with the activity of the peptides LL-37 and sheep leukocyte-derived SMAP-29 (37). Furthermore, in mixed populations of mucoid and non-mucoid *P. aeruginosa* strains, the mucoid bacteria producing EP alginate protected themselves and the non-mucoid bacteria from the action of LL-37 (26). On the other hand, the *Bordetella* polysaccharide (Bps) released by *Bordetella pertussis* interferes with the activity of LL-37 and the AMP polymyxin B (38). Likewise, the presence of Bps led to significantly higher loads of *B. pertussis* or *Escherichia coli* in the respiratory tract of infected mice, which could support that Bps are involved in the subversion of host defense mechanisms (38). Moreover, secretion of the exopolysaccharide Bps by a wild-type strain of *B. pertussis* conferred protection against the LL-37 peptide to a Bps-deficient mutant (38). In addition, colistin-susceptible *P. aeruginosa*, *E. coli*, and *Acinetobacter baumannii* were protected from lethal concentrations of colistin by the release of LPS (Fig. 1) (40).

On the other hand, CPs released by *K. pneumoniae*, *Streptococcus pneumoniae*, and *P. aeruginosa* interfere with the activity of polymyxin B by trapping it through electrostatic interactions (21). Cell-free capsule purified from a *Bacillus anthracis* capsulated strain protected a non-encapsulated mutant strain of *B. anthracis* against the activity of human β-defensins 2 and 3 (hBD-2 and hBD-3, respectively) and partially against the activity of human neutrophil α-defensin 1 (hNP-1) (39). *B. anthracis* releases capsule fragments under laboratory growth conditions and during host infection (39, 71, 72).

## SEQUESTRATION BY PROTEINS

Various proteins released by bacteria can also directly sequester AMPs or host-derived proteins that generate AMPs upon degradation (Fig. 1) (41, 42). For example, *B. cenocepacia* secretes BcnA, a lipocalin protein that sequesters polymyxin B (41). Under laboratory conditions, BcnA protected several bacteria from the action of polymyxin B, including *P. aeruginosa*, *Shigella flexneri*, *Salmonella enterica* serovar Typhi, *Acinetobacter lwoffii*, *A. baumannii*, and *Acinetobacter junii* (41). BcnA also protected *P. aeruginosa* from the action of polymyxin B in mice assays (41). Moreover, larvae of *Galleria mellonella* treated with BcnA were more susceptible to killing by *P. aeruginosa*, *K. pneumoniae*, *A. baumannii*, or *S. aureus* than larvae without the BcnA treatment, suggesting that BcnA protects these bacteria from the action of AMPs involved in the humoral immunity of *G. mellonella* (41). Consistently, *B. cenocepacia* lacking BcnA was poorly recovered from the hemolymph of *G. mellonella* larvae compared with the wild-type strain (41).

Lactoferrin-binding protein B (LbpB) is a surface-associated lipoprotein that has been involved in the protection against various AMPs, including LL-37, mouse AMP mCRAMP, synthetic peptides IDR1002 and IDR1008, human lactoferrin-derived peptide LfcinH (1–11), and bovine lactoferrin-derived peptide Lf (17–41) (42, 73). The production of LbpB is stimulated in iron-scarce environments (73, 74). In this stressful condition, *Neisseria meningitidis* produced and released LbpB via the NalP protease in the later growth phase (74). LbpB secreted by *Moraxella bovis* sequesters AMPs by forming extracellular gel-like structures (42). Interestingly, *M. bovis* LbpB may also act by binding and sequestering the host protein lactoferrin, preventing its degradation by proteases and consequently the release of lactoferrin-derived AMPs such as Lf (17–41) (42).

Some Group A *Streptococcus* (GAS) serotypes secrete the streptococcal inhibitor of complement (SIC) protein and the distantly related to SIC (DRS) protein, both of which are involved in the protection against several AMPs (43–45). *Streptococcus dysgalactiae* subsp. *equisimilis* secretes a SIC and DRS homologue protein, DrsG, which protects against the activity of the AMP LL-37 (46). The *S. aureus* secreted protein staphylokinase (Sak) and its proteolytically processed N-terminal variant, SakΔN10, bind to several AMPs such as hNP-1, hNP-2, LL-37, mCRAMP, melittin, pig-derived tritrpticin, and Lf (17–41) (47, 75, 76). Sak protected *S. aureus, E. coli*, *Streptococcus bovis*, and *S. epidermidis* against the action of hNP-1 and/or hNP-2 α-defensins (47). On the other hand, *Finegoldia magna* secretes the FAF protein that can sequester and neutralize the AMPs hBD-3 (48).

## DEGRADATION

Several bacteria secrete proteases that degrade different AMPs including human defense AMPs like defensins and LL-37 (Fig. 1; Table 1) (48, 61–67). The β-defensin hBD-1 is degraded by the proteases ZapA and ZmpB, secreted by *Proteus mirabilis* and *B. cenocepacia*, respectively (62, 63). The β-defensin hBD-2 and the β-defensin hBD-3 are degraded by the protease SepB secreted by *S. pyogenes* (48). The β-defensin hBD-3 is also degraded by gingipains proteases secreted by *Porphyromonas gingivalis* (66). On the other hand, the LL-37 peptide is susceptible to degradation by several secreted bacterial proteases, such as elastase produced by *P. aeruginosa*, gelatinase produced by *E. faecalis*, ZmpA produced by *B. cenocepacia*, aureolysin produced by *S. aureus*, metalloproteases produced by *B. anthracis*, SufA produced by *F. magna*, ZapA produced by *P. mirabilis*, and SepB produced by *S. pyogenes* (61–65, 67). Additionally, *Streptococcus suis* secretes the endopeptidase O (PepO) that degrades LL-37 and CRAMP; in the absence of PepO, *S. suis* was susceptible to LL-37 and CRAMP under laboratory conditions (23). In a murine bacteremia model, an *S. suis* mutant lacking PepO was susceptible to treatment with LL-37 (23). Furthermore, *S. suis* also secretes the cysteine protease ApdS that degrades LL-37; in the absence of ApdS, *S. suis* was more susceptible to free LL-37 and to human blood, macrophages, and neutrophils containing LL-37 (60).

On the other hand, secretion of the colistin-degrading protease Cdp by *Stenotrophomonas maltophilia* protected *P. aeruginosa* and *A. baumannii* against colistin when these bacteria were co-cultured in laboratory conditions, as well as protected *P. aeruginosa* against colistin in the *Drosophila melanogaster* infection model (27). These results suggest that physiological levels of the colistin-degrading enzyme released by *S. maltophilia* provide protection against colistin. This protection is clinically relevant because *S. maltophilia* and *P. aeruginosa* coexist in polymicrobial communities associated with human infections such as cystic fibrosis (27).

## MODIFICATION

Chemical modification of AMPs is another bacterial strategy for extracellular protection (Fig. 1). *P. gingivalis* secretes the citrullinating enzyme Porphyromonas peptidylarginine deiminase (PPAD), which citrullinates the arginine residues of the human lysozyme-derived LP9 peptide, thereby reducing its antimicrobial potency; citrullination converts arginine residues to neutral citrulline, thus affecting the net cationic charge of the LP9 peptide (22).

## SEQUESTRATION AND DEGRADATION THROUGH MEMBRANE VESICLES

In addition to biomolecules, bacteria also release membrane vesicles (BMVs), which are involved in protection against AMPs as well. BMVs are structurally and compositionally heterogeneous particles (diameter of ~40–400 nm) that have been involved in diverse biological functions such as protection from stressors, transfer of genetic material, virulence, nutrient acquisition, and detoxification (77). BMVs mediate protection against AMPs mainly by sequestering or degrading them (Fig. 1) (29, 78). The involvement of BMVs in the protection against AMPs has been reported for a variety of Gram-negative bacteria (29, 52–58, 79–81).

Production of BMVs can mediate temporary protection of *E. coli* against the peptide antibiotics polymyxin B and colistin by sequestering them through interactions with BMV-associated LPS (29). In addition, *E. coli*-derived BMVs protected this bacterium against the cathelicidin AMPs LL-37, chicken-derived AMP CATH-2, and porcine-derived AMP PMAP-36, presumably by acting as decoys (52). Under polymyxin B-induced stress conditions, *Vibrio cholerae* O1 El Tor produces BMVs containing the protein OmpT that binds the secreted protein Bap1, which in turn binds the AMP LL-37 (53). Furthermore, also in polymyxin B-induced stress conditions, *V. cholerae* mutants produced high levels of large BMVs that sequestered polymyxin B (59). Likewise, production of BMVs conferred protection to *A. baumannii* against polymyxin B under laboratory conditions and in bacteria infecting *G. mellonella* larvae (56). Interestingly, BMVs produced by *A. baumannii* also protected human gut microbiota from the antimicrobial activity of polymyxin B under laboratory conditions (56). Protection conferred by BMVs against polymyxin B has also been demonstrated for other bacteria such as *Acinetobacter oleivorans*, *Pseudomonas putida*, *P. aeruginosa*, *S. enterica*, and *K. pneumoniae* (55, 56, 58, 81, 82). Challenge of *K. pneumoniae* with polymyxin B or colistin induced a bulky release of small vesicles that sequester polymyxins through interactions with BMV-associated lipid A molecules (58). Moreover, the addition of BMVs from *K. pneumoniae* protected this bacterium against the polymyxin B challenge in precision-cut lung slices and in *G. mellonella* larvae (58).

On the other hand, *E. coli* produces BMVs containing proteases, such as Omptin, Do, D, and ClpA, which degrade the AMP melittin and thus confer protection against the activity of this peptide (78). Similarly, *V. cholerae* produces BMVs that contain the protease PrtV, which protects against the activity of the LL-37 peptide (68).

The production of BMVs is not restricted to Gram-negative bacteria; Gram-positive bacteria like *Mycobacterium tuberculosis*, *Streptococcus* sanguinis, *S. aureus*, *S. pneumoniae*, *S. suis*, *E. faecium*, *Clostridium perfringens, Listeria monocytogenes, B. anthracis,* and *Bacillus subtilis* can also produce BMVs (83). In response to the presence of the LL-37 peptide, GAS serotypes produced vesicle-like structures that bind to this peptide (84). Although sequestration and degradation of AMPs are the main mechanisms of protection exerted by BMVs, emerging evidence suggests that protection by BMVs may also be mediated by alternative mechanisms. For instance, BMVs generated by *P. aeruginosa* stimulated the production in this bacterium of the 2-heptyl-4-quinolone molecule, which sequestered the AMP LL-37 and thus prevented its antimicrobial activity (85). Other factors involved in resistance against AMPs have been also found within BMVs, like the LPS-modifying enzyme ArnT present in BMVs of *K. pneumoniae* (79); furthermore, the secreted PPDA enzyme that modifies the LP9 peptide has been found in BMVs from *P. gingivalis* (22, 86), raising the possibility that BMVs could also promote chemical modifications of AMPs. Interestingly, it was proposed that the production of BMVs could be a way to dispose of the bacterial membrane damaged by peptides, which also would mediate defense against AMPs (52).

The release of BMVs mediates collective defense against AMPs. *E. coli*-derived BMVs protected *P. aeruginosa* and *Acinetobacter radioresistens* in a dose-dependent manner against the colistin and melittin peptides (78). In addition, BMVs derived from *K. pneumoniae*, *E. coli*, *S. enterica* serovar Typhimurium, *S. enterica* serovar Typhi, or *Moraxella catarrhalis* protected different bacteria against polymyxin B (55, 57, 58).

Most studies about the protection mediated by BMVs were performed by testing purified BMVs. Whether BMVs provide protection against AMPs at physiological levels remains to be further investigated.

## COLLECTIVE PROTECTION

All releasome-associated defense mechanisms against AMPs would be expected to provide both individual and collective protection. In addition to the mechanisms already described, biofilm formation also mediates collective protection of bacteria against AMPs and other harmful agents (16, 87). A biofilm is a community of microorganisms encased in a self-produced extracellular matrix adhered to a surface; the extracellular matrix is composed of different molecules, such as extracellular DNA (eDNA), EPs, proteins, metabolites, among others (88). Treatment of *S. epidermidis*, *K. pneumoniae*, or *P. aeruginosa* biofilms with DNase I favored the antimicrobial activity of the synthetic peptide D-LL-31 against these bacteria, suggesting that eDNA plays a protective role in biofilms against AMPs (49). Protection against AMPs mediated by eDNA can occur directly or indirectly (50, 89). It has been shown that eDNA can bind and sequester hBD-2 and hBD-3 peptides (50, 51). Furthermore, eDNA has a chelating activity that creates a Mg^2+^-limited environment that triggers the expression of the AMPs resistance operon *arnBCADTEF-ugd*, which modifies the bacterial LPS and thus affects AMPs binding (89, 90). In addition to eDNA, EPs are also involved in protection against AMPs in the biofilm matrix (91, 92). Neutral and anionic EPs from the biofilm matrix of *K. pneumoniae* protected *K. pneumoniae* and *E. coli* against the bovine-derived peptides BMAP-27 and Bac7(1–35) (91, 92).

## CONCLUSIONS

Bacteria have several mechanisms to protect themselves against the activity of AMPs. Many of these mechanisms operate extracellularly and involve various molecules (such as proteins, phospholipids, polysaccharides, and extracellular DNA), particles (BMVs), or structures (biofilm formation) as part of the bacterial releasome, which can sequester, degrade, or modify AMPs. The extracellular defense of bacteria can provide not only individual but also collective protection against AMPs and thus would play an important role for survival in polymicrobial communities, such as those found in biofilms. A thorough understanding of the mechanisms mediating bacterial protection against AMPs could lead to the development of innovative AMP-based therapies. In support of this idea, several studies have shown that, in different animal models, pathogenic bacteria affected in AMPs resistance mechanisms showed attenuated infection (23, 30, 41). Moreover, inhibitors (α-tocopherol or menaquinone) of BcnA, a protein that mediates resistance against AMPs, reduced *P. aeruginosa* infection in *G. mellonella* larvae (41). Likewise, treatment with GWP-042, an inhibitor of the Rv2780 secreted enzyme that represses the expression of host AMPs, decreased *M. tuberculosis* infection in mice (30).
